# Integrating optimal patient-centered care: patient- and family-reported experiences after complex treatment for rare diseases

**DOI:** 10.1007/s12519-026-01089-4

**Published:** 2026-09-19

**Authors:** Frederiek L. Tijssens, Philippine Kies, Lissy de Ridder, Anne P. J. de Pagter, Jeanine M. M. van Klink

**Affiliations:** 1 Department of Pediatrics, Willem-Alexander Children’s Hospital, Leiden University Medical Center, Albinusdreef 2, Postal Zone J-6-S, 2300 RC Leiden, The Netherlands; 2 Department of Cardiology, Leiden University Medical Center, Leiden, The Netherlands; 3 Division of Stem Cell Transplantation, Department of Pediatrics, Willem-Alexander Children’s Hospital, Leiden University Medical Center, Leiden, The Netherlands; 4 Division of Medical Psychology, Department of Psychiatry and Psychology and Division of Neonatology, Department of Pediatrics, Willem-Alexander Children’s Hospital, Leiden University Medical Center, Leiden, The Netherlands

**Keywords:** Care pathway, Integrated delivery of healthcare, Patient-centered care, Rare diseases, Value-based healthcare

## Abstract

**Background:**

Although life expectancy after complex treatments for rare diseases during childhood has increased, long-term medical, psychosocial and societal impacts remain for children and their families. Evaluating patient and family experiences is essential for the development and optimization of integrated patient-centered, value-based care pathways. This study aims to evaluate patient- and family-reported experiences across three follow-up care pathways for complex treatment of rare diseases.

**Methods:**

This cross-sectional study was conducted among patients/families in follow-up care after (1) fetal and/or neonatal intensive care treatment; (2) pediatric hematopoietic stem cell transplantation for non-malignant conditions; and (3) cardiac intervention for congenital heart disease. A combination of validated patient-reported experience measures (PREMs) was used to capture experiences with complete integrated care pathways regarding information provision, integrated care collaboration, communication and comprehensive care. Descriptive statistics were used to analyze quantitative ratings, whereas free-text responses were analyzed using thematic analysis.

**Results:**

Between April 2024 and June 2025, 70 families completed the PREMs. Likert-scale ratings, as well as an analysis of 286 free-text responses, revealed positive evaluations of relationship building, the humanistic approach and communication across all pathways. The identified areas of improvement varied across care pathways and included comprehensive (long-term) information provision and screening and support for the psychosocial impact on patients as well as their families.

**Conclusion:**

Through shared learning across care pathways, insights from patient and family experiences guide patient- and family-centered, value-based care pathway optimization to ultimately improve outcomes and quality of life for children and their families.

**Graphical abstract:**

**Figure:**
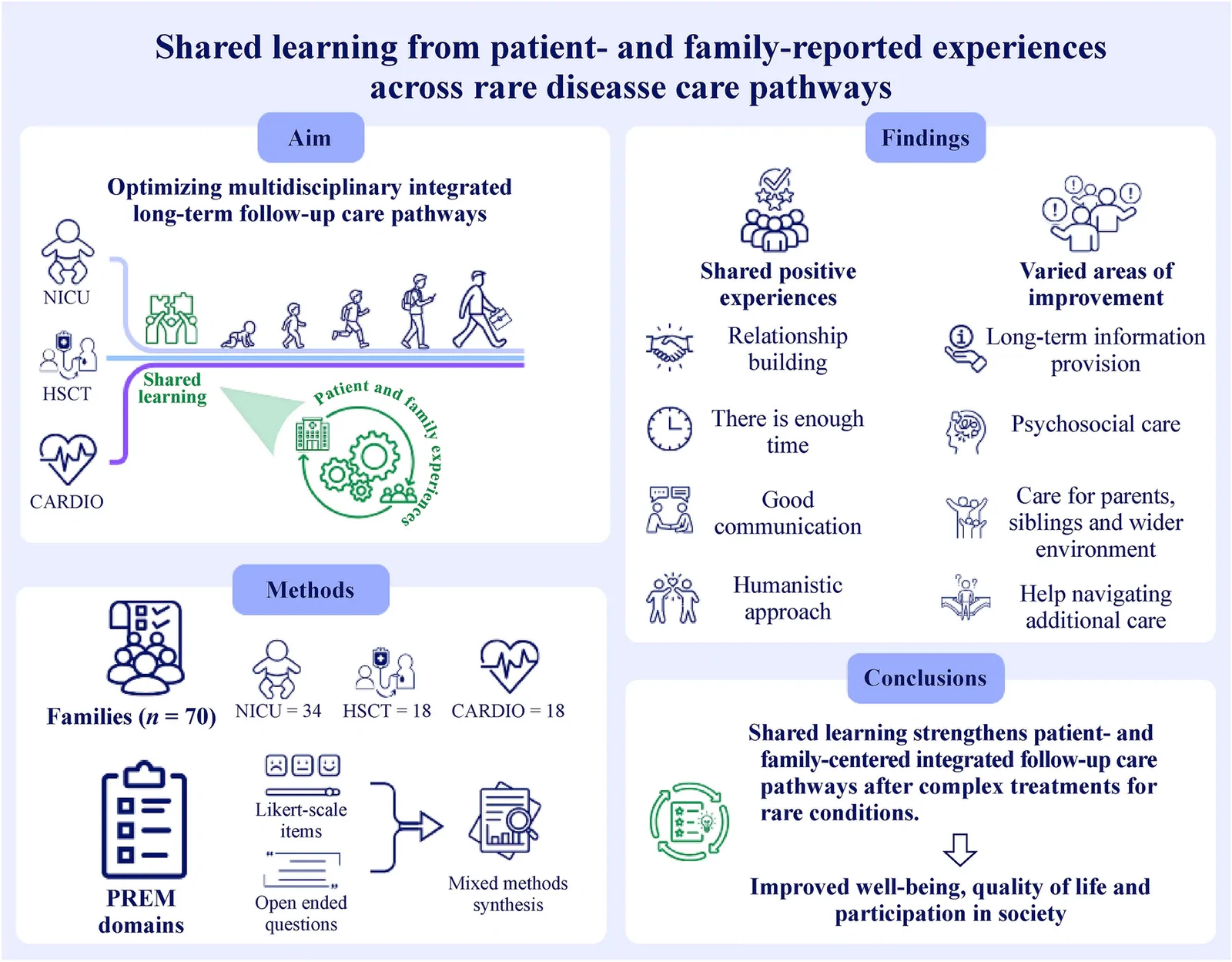

## Introduction

With medical advances, survival and life expectancy after complex treatment for rare diseases during pregnancy, infancy or childhood continue to increase [1–4]. Although treatments aim to cure the initial disease, many children and their families remain vulnerable to acute and long-term consequences [3–5], which are often insufficiently addressed [6–8]. This impact extends beyond medical outcomes and includes psychological, neurocognitive, relational and societal consequences [8–10]. These factors may persist throughout life, affecting quality of life and participation in society [11–13].

Research suggests that children treated with complex treatments for rare conditions face similar long-term outcomes, including neurocognitive difficulties [14–16], attention and stimulus processing problems [9, 11, 17, 18], and a need for extra support at school [19–22]. However, effective prevention, early detection, screening and management of these outcomes often remain insufficiently embedded and thus fragmented in many care pathways for complex treatments [7, 23–25]. This highlights the need for dedicated “integrated follow-up care pathways”, such as structured long-term follow-up providing screening and specialized care for the health and well-being of patients and their families. These pathways require multidisciplinary collaboration both within and beyond the hospital, as families’ care needs vary across developmental stages. The optimal integration of care elements across disciplines and age groups is therefore essential [26].

The development of such pathways requires a person-centered approach, as reflected in patient- and family-centered care (PFCC) and value-based healthcare (VBHC) [27, 28]. In pediatrics, PFCC promotes partnership and collaboration with patients and families by incorporating their experiences, preferences and values into care processes [28–31]. In doing so, care can be tailored to families’ needs [29, 30]. Similarly, VBHC recognizes patient and family experiences among three key healthcare outcomes [27, 32, 33]. Both prioritize integrating patient and family experiences as fundamental to the quality of care [28].

These experiences are typically captured through patient-reported experience measures (PREMs), which evaluate care processes, including information provision, communication with healthcare professionals (HCPs), shared decision-making and consultation timing [34–37]. PREMs facilitate patient engagement, personalized care and improved communication [30, 35–37]. Moreover, positive healthcare experiences are associated with better treatment adherence, reduced healthcare utilization and improved clinical and patient-reported outcomes [38–41]. Above all, systematically evaluating PREMs provides essential insights into what patients and families value, identifies key areas for quality improvement and enables benchmarking of care pathway quality from patient and family centeredness [34, 35, 38, 42–45]. However, previous studies on PREMs in pediatric care have mainly assessed a single consultation or inpatient phase, particularly in acute or chronic care settings [46–49], rather than the complete patient journey of an integrated follow-up care pathway [26, 47, 50]. Moreover, despite shared challenges, no studies have explicitly compared patient experiences across multiple care pathways for complex treatments to accommodate joint improvement initiatives.

Therefore, this study aims to compare patients’ and families’ experiences with integrated follow-up care pathways after complex treatment for rare diseases in three nationwide tertiary pediatric referral centers for fetal therapy and neonatal intensive care, hematopoietic stem cell transplantation (HSCT) for non-malignant conditions and cardiac intervention for congenital heart disease (CHD). Through knowledge sharing across care pathways, overarching building blocks for an integrated PFC-VBHC pathway for highly complex treatments can be identified to ensure optimal patient journeys and comprehensive care.

## Methods

### Study setting and participants

This cross-sectional study at the Willem-Alexander Children’s Hospital of the Leiden University Medical Center (LUMC) is a collaboration between three existing expertise centers: (1) fetal therapy and/or treatment at the neonatal intensive care unit (NICU); (2) pediatric HSCT for non-malignant diseases; and (3) cardiac intervention in the first year of life for CHD. All three centers run an integrated follow-up care pathway with periodic risk-based screening on the basis of patient-reported outcome measures. All families receive structured follow-up appointments with their specialist (neonatologist, hematologist or cardiologist) at intervals of every 1, 2 or 3 years, depending on the life stage. As the three pathways are oriented toward similar (long-term) outcomes, they work in similar multidisciplinary care networks. Families are regularly offered consultations with a psychologist, social worker, physiotherapist or other HCPs on indication. There are some organizational differences, such as the duration of follow-up. More information on the present follow-up care pathways within the LUMC is provided in Supplementary Material 1.

Between April 2024 and June 2025, patients and/or their caregivers were eligible if they had previously had at least one follow-up appointment within the above-described care pathways. For this study, people who did not speak Dutch were not eligible. Participants were recruited via two routes: first, the study invitation was included in the mailing for the communal conference day of the three care pathways and sent to all attending families (*N* = 96). Second, a digital newsletter including the study invitation was sent to all patients who received follow-up care from 2021 to 2023 [*n* = 1908 NICU; 187 HSCT; 834 cardiology (CARDIO)]. Finally, a bulletin of this study was shown on the waiting room information screens. Everyone who registered (regardless of the recruitment strategy) was sent a study information letter and informed consent form. The study was conducted in accordance with the Declaration of Helsinki [51] and was approved by the institutional review board of the Medical Ethics Committee Leiden The Hague Delft (NL20.181).

### Patient-reported experience measure

To assess families’ experiences with all integrated follow-up care pathways after complex treatments regardless of the underlying condition, the multidisciplinary research team of adult and pediatric medical specialists, psychologists, nurse experts and researchers adapted a validated general childcare PREM of the Dutch Integrated Childcare Association [52]. The original instrument served as a structural foundation: its core domains were retained, while adaptations were made to better fit the study’s aim to evaluate the complete patient journey within the current complex practice of the integrated care pathways. As no single PREM fully captured this, adaptations were based on several existing cocreations of PREMs from comparable patient populations [35–37, 53–57]. Items were added to reflect what is important to the present patient population, whereas other items were deleted if they addressed care elements not present in the current care pathways. After family representatives pilot tested the PREM on usability, clarity and completeness [57, 58], it was finalized in a consensus meeting. The PREM includes four demographic questions covering the treatment received, patient age and gender and respondent type (caregiver, patient, etc.). It also includes 21 Likert-scale questions (ranging from 1—totally disagree to 5—totally agree) addressing care aspects such as information provision or comprehensive care provision. Four open-ended questions were answered on the basis of their Likert-scale responses, along with one final open-ended question for additional comments. A version with adjusted, age-appropriate language was created for 12- to 18-year-old patients. The PREM (translated from Dutch) is provided in Supplementary Material 2.

### Study procedure

After signing the informed consent form, participants were invited to complete the PREM by post (for conference day attendees) or digitally (for those registered through the newsletter or bulletin). The caregivers of twins received one questionnaire for both children jointly, as they had joint follow-up appointments. Families with a ≥ 12-year-old child were sent one PREM for the child and one for the caregiver(s), since studies have reported consistent differences between child- and proxy-reported experiences [37, 54]. PREM responses were encrypted into Castor-EDC before further analysis.

### Analysis

Descriptive statistics were provided using SPSS (version 29.00). Participant demographics and question ratings were summarized per care pathway using medians and ranges for continuous variables and frequencies and percentages for categorical variables. To evaluate the most positively and negatively rated Likert-scale questions, the top- and bottom-box scores were calculated per question. An item’s top-box score is the joint proportion of respondents selecting response options “5—strongly agree” or “4—somewhat agree”. The bottom-box score reflects the proportion of respondents choosing either “1—strongly disagree” or “2—somewhat disagree”. Items with higher top-box (5 or 4) scores were considered the most positively evaluated. High bottom-box (1 or 2) scores indicated more negative evaluations and were used to clearly distinguish between true negative ratings and merely low positive or high neutral ratings.

The free-text answers were used to deepen the understanding of why aspects of care were experienced as positive or negative in addition to the quantitative data. Themes, sub-themes and supporting quotes were chosen following the six stages of thematic analysis by Braun and Clarke using Atlas.ti (version 24) [59].

## Results

### Participant characteristics

**Fig. 1:**
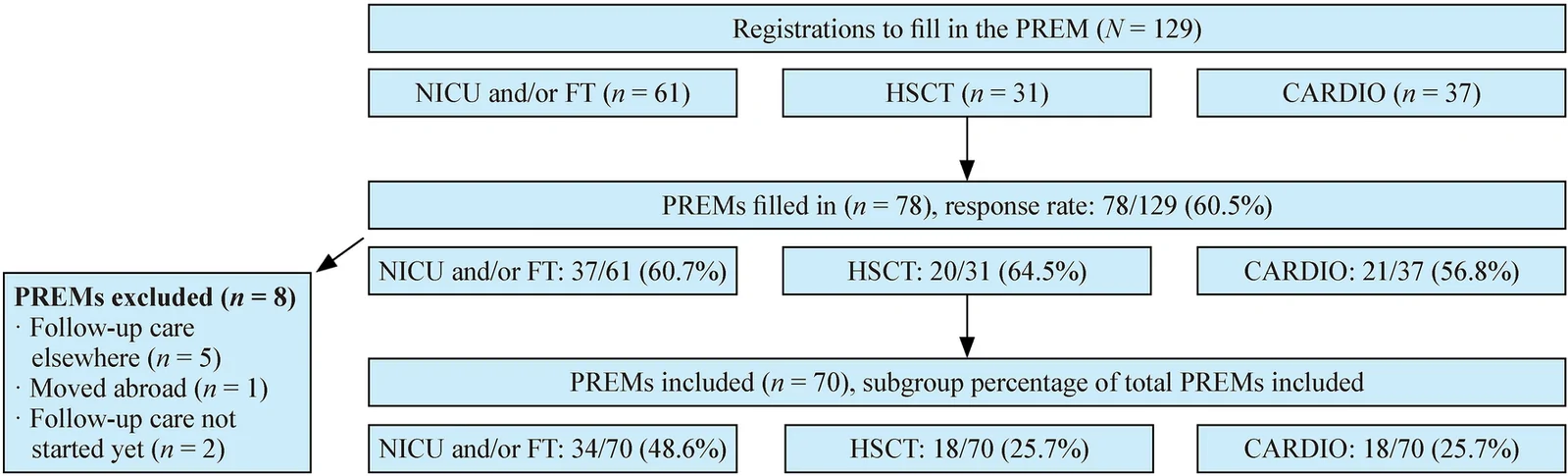
Flowchart of the included respondents. *PREM* patient-reported experience measure, *NICU* neonatal intensive care unit, *FT* fetal therapy, *HSCT* hematopoietic stem cell transplantation, *CARDIO* cardiology

Of the 129 registered participants, 78 (60.5%) completed the PREM. Of these, 70 (89.7%) responses were suitable for analysis, as illustrated in Fig. 1. Thirty participants were recruited through the patient day and 40 through the newsletter or bulletin. Patient characteristics are summarized in Table 1. Eleven of the 70 (15.7%) PREMs were completed by caregivers of children who had had two complex treatments, such as a cardiac intervention and a HSCT. They were asked to report on their primary follow-up care pathway only.

**Table 1:** Demographics of the participants

Demographic	NICU PREMs (*n* = 34); children (*n* = 42)	HSCT PREMs (*n* = 18); children (*n* = 19)	CARDIO PREMs (*n* = 18); children (*n* = 19)	Total PREMs (*N* = 70); children (*n* = 80)
Twins, *n* (% of PREMs)^a^	8 (23.5)	1 (5.6)	1 (5.6)	10 (14.3)
Child gender, *n* (% of children)				
Male	24 (57.1)	9 (47.4)	11 (57.9)	44 (55.0)
Female	17 (40.5)	9 (47.4)	8 (42.1)	34 (42.5)
Non-binary	1 (2.4)	1 (5.3)	0 (0.0)	2 (2.5)
Child age (y), median (range)	4.0 (0–12)	10.5 (2–32)	9.5 (1–20)	6.0 (0–32)
Filled in by, *n* (% of PREMs)^b^				
Both parents	12 (35.3)	5 (27.8)	3 (16.7)	20 (28.6)
Father	1 (2.9)	1 (5.6)	2 (11.1)	4 (5.7)
Mother	21 (61.8)	8 (44.4)	10 (55.6)	39 (55.7)
Guardian	–	1 (5.6)	1 (5.6)	2 (2.9)
Child (≥ 12)	–	2 (11.1)	–	2 (2.9)
Child (≥ 12) + parent/guardian	–	1 (5.6)	2 (11.1)	3 (4.3)
Indication for inclusion in care pathway, *n* (% of children)^c^	6 (14.3); twin–twin transfusion syndrome	11 (57.9); bone marrow failure	1 (5.3); mild CHD	
	27 (64.3); premature birth	5 (26.3); immunodeficiency	9 (47.4); moderate CHD	
	4 (9.5); perinatal asphyxia	3 (15.8); hemoglobinopathy	9 (47.4); severe CHD	
	5 (11.9); perinatal brain injury			
Time since complex treatment (y), median (range)	4.0 (0–12)	4.0 (2–24)	9.5 (1–20)	4.75 (0–24)

*PREMs* patient-reported experience measures, *NICU* neonatal intensive care unit, *HSCT* hematopoietic stem cell transplantation, *CARDIO* cardiology, *CHD* congenital heart disease. ^a^The number of children is greater than the number of PREMs, as caregivers of twins filled in one joint PREM; ^b^there are two families in which both the parent and the child filled in a PREM independently of each other; ^c^five of the children in the NICU group received NICU treatment for two conditions (i.e., premature birth and asphyxia). According to the ESC Guidelines, nine of the children in the CARDIO group have multiple CHDs, for which the most severe CHD is used for classification [60]. “–” no data

### Patient-reported experience measure: Likert-scale questions

**Fig. 2:**
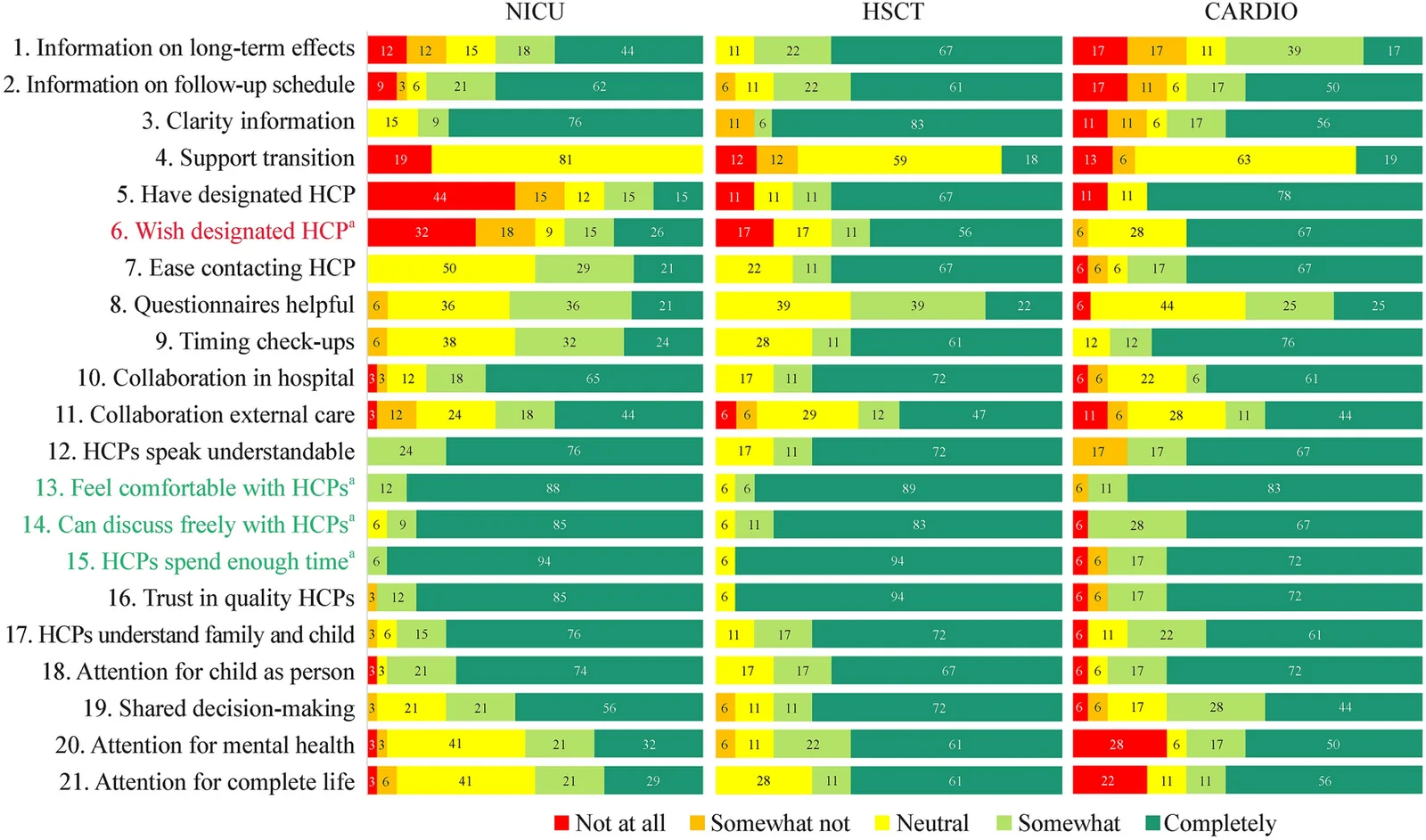
Percentages of Likert-scale ratings per follow-up care pathway. *NICU* neonatal intensive care unit, *HSCT* hematopoietic stem cell transplantation, *CARDIO* cardiology, *HCPs* healthcare professionals. ^a^The questions written in green or red have received the highest top- or bottom-box scores in multiple care pathways

The ratings of the Likert-scale questions are summarized per care pathway in Fig. 2. For all three pathways, most response distributions are left skewed toward a positive assessment.

For NICU respondents, Q15 (HCPs spend enough time), 13 (feel comfortable with HCPs) and 12 (HCPs speak understandably) received the highest percentage of the top box, 100%. The highest bottom-box percentages were observed for Q6 (wish for designated care provider) at 58.8% and Q5 (have a designated care provider) at 50.0%. This shows that half of the respondents do not have a designated care provider, yet more than half indicated that they do not wish to have one. This may reflect that families feel adequately served by their existing healthcare contacts. This finding is supported by the overarching top-box score of feeling comfortable with the HCPs and the qualitative findings in which families expressed trust in the quality of the HCPs and positive experiences with reaching the right HCP when needed.

For HSCT respondents, Q13 (feel comfortable with HCPs) had a top-box score of 94.5%, and Q14 (can discuss concerns and wishes freely with HCPs), 15 (HCPs spend enough time) and 16 (trust in quality HCPs) had top-box scores of 94.4%. The highest bottom-box scores were found for Q6 (wish for designated care provider) at 23.6% and Q4 (given support for adult care transition) at 16.7%. Given that the median age of the patients was 10.5 years, with a minimum of 2 years, the transition to adult care was not applicable to most participants.

For the CARDIO respondents, the aspects of care that were experienced the most positively were Q13 (feel comfortable with HCPs) and 14 (can discuss concerns and wishes freely with HCPs), with 94.4% top-box scores for both. The highest percentage of bottom-box scores was for Q20 (HCPs pay attention to children’s mental health), with 33.3%, followed by Q1 (information on long-term effects) and Q21 (HCPs pay attention to complete living situations, e.g., family and friends, school, and home functioning), with a bottom-box score of 27.8%.

In summary, three aspects of care were the most positively experienced items in multiple care pathways: Q13 (feel comfortable with HCPs) in all three groups and Q15 (HCPs spend enough time) and Q14 (can discuss concerns and wishes freely with HCPs) in two of the three groups. With respect to the aspects of care that were mostly not experienced by the respondents, there is more disparity between the care pathways. Only Q6 (wish for designated care provider) had one of the highest bottom-box scores in two of the three groups.

### Patient-reported experience measure: free-text responses

Of the 286 free-text responses analyzed across the five open-ended questions, six themes were identified: (1) information provision; (2) care communication and coordination; (3) humanistic; (4) comprehensive; (5) additional care; and (6) logistics. Table 2 provides an overview of how the themes are defined, what codes they encompass and extra exemplary quotes.

**Table 2:** Themes, codes and subcodes of the free-text responses of the patient-reported experience measure with illustrative quotes

Theme and sub-themes		Number of comments (*N* = 286)	Definition of theme and illustrative quotes
Information provision		36 (12.6)	How people were informed, whether the information was clear and sufficient and what topics were missing
+	Sufficient information	6	“During the appointments, we received sufficient explanation and were able to ask all our questions. Additionally, we were given or sent written information, which made the information complete for us” (mother of a 4-y-old; NICU) “Clear information was provided” (mother of a 2-y-old; HSCT)
	Clarity	11	
	Verbal and written	3	
	Total	20 (7.0)	
−	Long-term consequences missing	12	“Not much was said about ‘later’. Only that she most likely will not need any major open-heart surgeries anymore” (mother of a 1-y-old; CARDIO) “Provide a clearer picture of the follow-up appointments and give progress updates of studies we’ve participated in” (parents of a 3-y-old; NICU) “I find the information from doctors difficult to understand” (patient, 20 y; CARDIO)
	Clarity	4	
	Total	16 (5.6)	
Care communication and coordination		75 (26.2)	The communication and cooperation between all parties involved in the care process, including the family, the child, all HCPs involved and other care institutions
+	HCP-family	9	“It is always clearly explained why a particular test is being done and what certain results mean” (parents of a 4-y-old; NICU) “The general practitioner mentioned that the collaboration is smooth in regard to information transfer” (mother of a 9-y-old; HSCT) “Our cardiologist and pediatrician work together” (mother of a 16-y-old; CARDIO) “We always get to speak with the doctor who, with their expertise, is best able to answer the question at hand” (parents of a 1-y-old; NICU)
	Intra-hospital	4	
	Inter-organizational	10	
	Accessibility for questions	18	
	Total	41 (14.3)	
−	HCP-family	8	“My husband is a doctor. Sometimes, because of that, things are discussed in medical terms, while I (the wife) don’t have a medical background. This makes it harder for me to understand at times” (parents of 2-y-old; NICU) “Not all HCPs are equally good at communicating with my child. My son has developed a fear of needles, and this was not clearly communicated from the ‘blood draw clinic’ to the cardiologist” (mother of 9-y-old; CARDIO) “It’s not easy to get in touch with my HCP” (patient with parent, 19-y-old; CARDIO) “Your child is monitored for a longer period after hospitalization. After an intensive hospital stay, it is comforting to maintain close contact with the hospital, as GPs and the health clinic often lack sufficient knowledge of the consequences after a prolonged hospital stay” (parents of a 1-y-old; NICU)
	Intra-hospital	10	
	Inter-organizational	7	
	Transfer of knowledge to other care settings	3	
	Accessibility for questions	6	
	Total	34 (11.9)	
Humanistic		65 (22.7)	Everything related to the approach of the patient and family that treats them with kindness, dignity and respect needed for good relationship building
+	Relationship building	5	“It feels like home, she can be herself” (parents of a 10-y-old; CARDIO) “We are being listened to, and we have a lot of trust in the pediatric cardiology department” (mother of 10-y-old; CARDIO) “The doctors listen to the parents' side. We feel taken seriously. The doctors give us the sense that our perspective on our son's health is valuable in providing the most appropriate and necessary care” (parents of a 1-y-old; NICU)
	Active listening	15	
	Shared decision-making	7	
	Compassionate care	15	
	Trust in HCPs	12	
	Total	54 (18.9)	
−	Relationship building	6	“Some staff members do not know the whole story and could empathize a bit more” (mother of a 1-y-old; CARDIO) “Everyone also has a preference for a nurse or doctor. The connection with one is stronger than with another” (parents of a 6-y-old; HSCT)
	Being a “case”	5	
	Total	11 (3.8)	
Comprehensive care		29 (10.1)	Care that considers not only the medical health of the patient, but all aspects that contribute to quality of life, such as psychosocial well-being, neurocognitive outcomes and family functioning
+	Attention to psychosocial aspects	6	“They definitely ask how the parents are doing, as they need to be stable for the child's good health. We had a positive experience with that” (parents of a 6-y-old; HSCT) “They often ask how things are going, both with the child and with us. They truly act on this and offer help (parents of 2-y-old; NICU) “The cardiologist took our living conditions into full consideration” (mother of a 12-y-old; CARDIO)
	Attention to child’s surrounding systems	7	
	Total	13 (4.5)	
−	Attention to psychosocial aspects	8	“We ourselves asked for advice regarding the school; the psychologist did not bring this up on their own” (parents of a 7-y-old; NICU) “The focus is still mainly on the medical condition, with little attention given to mental health” (mother of a 6-y-old; CARDIO) “Attention to the mental aspect for parents (both fathers and mothers), especially sometime after birth” (mother of a 4-y-old; NICU)
	Attention to child’s surrounding systems	8	
	Total	16 (5.6)	
Additional care		19 (6.6)	Experiences with finding additional and/or supportive care (such as additional consultations, receiving support at school, referrals to other healthcare institutions) that extend beyond the scope of the care pathway
+	Support finding additional care	5	“We can always discuss mental health with both the cardiologist and pediatrician and ask for their help in referring to other support (psychologist, etc.)” (mother of a 16-y-old; CARDIO)
	Total	5 (1.7)	
−	Difficulty finding additional care	10	“Broader support in case of any developmental issues with the child” (mother of 3-y-old twins; NICU) “It would be great if more attention were given to aftercare for children with BPD” (both parents of a 5-y-old; NICU) “We need a longer follow-up. Why does it end after 8 years?” (parents of 9-y-old twins; NICU). “When we were allowed to go home, we were let go. If there was something, we were told to call, but to this day, I miss having a person to turn to for all the questions you have as a parent” (mother of a 6-y-old; CARDIO)
	Support transition after inpatient period	4	
	Total	14 (4.9)	
Logistics		35 (12.2)	All comments about specific areas related to the planning, location, time and accessibility of the hospital or the HCP
+	Sufficient time	9	“Time is taken for our daughter during the check-ups, and if we have questions in between, we can easily ask them” (mother of a 9-y-old; HSCT) “The follow-up appointments always run smoothly” (parents of a 4-y-old; NICU)
	Appointments run smoothly	3	
	Total	12 (4.2)	
−	Insufficient time	5	“To truly take the time to answer questions, not during the ultrasound and while keeping the little one still/quiet, but just afterwards, taking the time to ask and answer questions calmly” (mother of a 3-y-old; CARDIO) “Some conversations are fine to have with the child present, while others are better discussed between the parents. Now the child is always present, and he interprets our concerns more seriously, while we want to keep things light-hearted for him” (mother of 11-y-old twins; NICU) “Appointment confirmation letters often arrive too late” (both parents of a 2-y-old; HSCT)
	Care planning	9	
	Location issues	2	
	Correspondence or questionnaires	7	
	Total	23 (8.0)	

*NICU* neonatal intensive care unit, *HSCT* hematopoietic stem cell transplantation, *CARDIO* cardiology, *HCP* healthcare provider, *BPD* bronchopulmonary dysplasia, *GP* general practitioner. “ + ” positive, “−” negative

#### Information provision

Free-text responses further confirmed that people often missed information, specifically on the long-term comprehensive consequences; however, it was helpful to receive both explanations during visits and information through brochures: “At the time, we were informed by doctors and nurses about possible consequences, also through brochures. However, these were the ‘major’ consequences, as I remember them. Less known is disrupted sensory processing and its signs. I think especially the latter would be important to mention” (mother of a 6-year-old; NICU).

#### Care communication and coordination

In the care pathway, many HCPs have to collaborate across multiple organizations. What seems to be most important is that the families speak to the appropriate HCP, who is well informed about their situation and that this process is transparent to the family: “I’m very satisfied with how information was shared with other HCPs (hospitals). The information that was shared was also visible to me, which is great” (mother of a 1-year-old; NICU). Most families find it easy to get in contact when they have questions, whereas some say it is not. This is an important barrier to finding appropriate care: “It’s very easy reach the HCPs within LUMC, but outside LUMC, it’s a lot of hassle through the general practitioner (GP). We are told (by LUMC HCPs) where to go, but then completely get left to our own devices” (mother of 11-year-old twins; NICU).

A barrier to cross-organizational communication is a lack of knowledge about complex treatments and their consequences in primary and secondary care. This is due to the absence of a unified information transfer system and limited expertise outside the specialized center to interpret the child’s medical history: “Especially when our child was still being monitored at the child health clinic, we strongly felt that the pediatrician in place was not well informed about his heart condition. The LUMC did send the information, but the knowledge was lacking at the health clinic. This could be improved, as it sometimes causes a lot of misunderstanding during these appointments” (mother of a 6-year-old; CARDIO). “The GP is often not aware of what’s happening at the LUMC. I always have to tell my story again” (patient, 28 years old; HSCT).

#### Humanistic

The respondents reflected that when the HCPs actively listened and worked together with the caregivers as equal partners, they felt taken seriously and understood: “The feeling that you’re working together to care for your child is a very comforting feeling” (mother of a 9-year-old; HSCT). Important prerequisites to building such mutual understanding and trust are kindness, empathy and compassion. Altogether, this humanistic approach helps us to forge a positive partnership for family-centered care and shared decision-making: “What I mostly remember is the empathy and clarity with which our sons’ diagnosis was communicated to us. It was so comforting to be involved in a situation where as parents, you’ve lost all control and to hear that the doctors were consulting with each other and genuinely wanted to understand our story. That bit of humanity toward us as a family definitely helped us process our story and allows us to look back on some very valuable moments” (mother of 5-year-old twins; NICU). On the other hand, a few families mentioned less positive experiences: “More attention is given to my child as a medical ‘object’ rather than as a person, in my opinion” (mother of a 4-year-old; NICU).

#### Comprehensive

The respondents stressed the importance of attention to and support for the psychosocial impact of a complex treatment on the child but also the parents, siblings and other surroundings, such as school: “I’m happy with the improvements on the mental health front, but there’s still room for improvement. More attention should be given to the whole family and the impact of a hospital stay on the family. More preventive measures, such as trauma processing or prevention (for example, a ‘recovery kit’ after treatment with similar materials for at home)” (parents of a 1-year-old; CARDIO).

#### Additional care

The respondents mentioned that they wanted more extensive care with more guidance in finding appropriate support beyond the care pathways. People described having difficulty navigating the healthcare landscape or feeling “let go of” and “lost” after the inpatient period: “Within the walls of LUMC (nine weeks of treatment and subsequent visits), everything was great. However, the recovery took much longer, and support, guidance, and assistance for parents, school, family, and friends were lacking. That has an impact on a child” (father of a 14-year-old; HSCT).

### Logistics

Logistics include comments on consultation timing and location in alignment with care needs (close to home when possible), having enough time and consultations running smoothly: “Appointments go smoothly, although they sometimes take a long time. It’s nice that everything is scheduled on the same day” (mother of a 9-year-old; CARDIO).

## Discussion

This study is the first to evaluate patient and family experiences across three integrated care pathways following complex treatment for rare diseases, deliberately crossing the boundaries of siloed, organ-specific disciplines. By capturing the complete patient journey rather than isolated clinical encounters and by integrating all multidisciplinary care within each pathway, this study moves beyond traditional disease-specific frameworks. This not only identifies overarching building blocks for PFCC but also creates unique opportunities for shared learning and synergy across conditions that are rarely studied together.

The results of the quantitative data revealed that most families positively evaluated follow-up care. Factors that contributed to positive experiences were largely equivalent across care pathways. These were “to feel comfortable, have enough time and feel free to discuss all concerns and wishes with HCPs”. In contrast, the points for improvement differed across the three care pathways, showing opportunities for shared learning from one another’s care organization.

The qualitative data further emphasized the importance of a humanistic approach and relationship building among patients, family and HCPs. Both the free-text responses and the bottom-box scores pointed to two key areas for improvement. First, information provisions, especially regarding long-term consequences, were missed. Second, families mentioned a wish for more comprehensive screening and care for neurocognitive development, psychosocial well-being and the practical impact on the child, parents, siblings and wider surroundings such as school. An additional area of improvement not captured by the Likert-scale questions was the desire for more guidance in finding additional care outside the hospital. In line with similar studies, families described spending considerable time navigating the healthcare network and struggling to find the appropriate care for their additional support needs by themselves [24, 61, 62]. This is costly, since poorer psychological well-being among children and parents is associated with greater care consumption [63–65] and developmental delays [66–69].

Taken together, these results are in line with those of previous international studies on complex diseases [38, 44, 61, 62], as well as studies on pediatric cancer [70, 71]. The importance of family-HCP interactions, including providing good information and taking the time to answer questions, was among the main determinants of positive experiences in pediatric surgical care [38]. Furthermore, feeling understood and taken seriously in the care for your child were determinants of positive experiences in two neonatal care studies in Scandinavia [44, 62] and one study in pediatric hemophilia care in the Netherlands [61]. These studies also stress the importance of better information provision and more comprehensive care [38, 61, 62]. Interestingly, the field of (pediatric) oncology demonstrates that structured integrated long-term follow-up care is feasible at scale [72–74]. Through standardized European outcome sets and harmonized follow-up guidelines, multidisciplinary teams can systematically monitor physical, psychosocial and neurocognitive consequences. Insights from this field could inform the development of coordinated multidisciplinary care in other complex pediatric care pathways.

From a life course perspective, the identified follow-up needs and experiences with care are likely shaped by the timing of care relative to both the child’s developmental stage and the time since treatment [75, 76]. In the early phase, families are often in an acute adjustment phase, in which they manage high levels of uncertainty and distress. Care needs mainly center around emotional support, understanding the condition, practical caregiving and daily life adjustments. Research reports that current care for these needs is often lacking or, on the other hand, risks information overload [77–80]. These findings highlight the need for stage-specific patient- and family-centered practical and psychosocial support to foster caregiver resilience, tailored communication and structured information provision.

This perspective reinforces the importance of viewing follow-up care as a longitudinal process. Child development is a cumulative process in which unmet neurocognitive and psychosocial needs at early developmental stages can compound over time and shape future health trajectories [75, 76]. Follow-up services should anticipate that support needs evolve over time and provide timely access to appropriate expertise rather than relying on reactive referrals from isolated clinical encounters. In this context, families’ wishes for better information about long-term consequences and more comprehensive psychosocial care likely reflect retrospective recognition of unmet care needs in their patient journeys. Future research should examine how follow-up experiences and unmet needs vary across developmental stages and time since treatment and how care pathways can better anticipate these changing priorities.

A strength of this study is the combination of quantitative and qualitative data. The Likert-scale items enable benchmarking to evaluate quality improvement initiatives. Additionally, the free-text responses allow for a comprehensive understanding of what contributes to patients’ and family members’ experiences. Although objective questions about whether certain events did or did not occur are useful, they do not fully capture which aspects patients and their families value most. Some adjustments to the PREM could yield even richer information about what contributes to the quality of care and positive experiences for patients and families.

Moreover, there may be some recruitment and selection bias, as involvement or affinity with the hospital could have positively or negatively influenced the results and their generalizability. Second, the exclusion of families who do not speak Dutch limits generalizability and may have biased the findings toward more positive experiences. A language barrier can directly affect several of the aspects evaluated in this study, such as communication with HCPs, understanding of information provision, and access to support resources. Although the questionnaire was deliberately written at the B1 language level and 80%–90% of the people in the Netherlands have this level of language proficiency [81, 82], individuals with lower Dutch proficiency were excluded. This also applies to low digital literacy, as all routes of recruitment involved at least one online step. In light of the PFC-VBHC, the experience of all patients, in particular of the “hard-to-reach” population, is important for in-depth insights for care pathway improvement. These families may face more barriers to care and experience greater societal and psychosocial vulnerability. To ensure optimal equity and inclusivity in the future, face-to-face or postal recruitment methods could help reach those with limited digital literacy, while questionnaires translated into multiple languages could improve inclusivity among non-Dutch-speaking families.

In conclusion, the present study provides comparative insights into patient and family experiences that can be used to optimize integrated PFC-VBHC pathways after complex treatment. This was done by crossing boundaries of the original condition and involving all (pediatric and adult) disciplines. Institutions should (continue to) prioritize a humanistic approach and good relationship building. Furthermore, long-term psychosocial consequences beyond medical outcomes should be systematically addressed in care so that families no longer feel “lost” in finding appropriate supportive care. Key facilitators are better information provision and the formation of a strong, comprehensive care network. Structurally embedding these aspects in care pathways is essential for high-quality PFC-VBHC. This highlights the urgent need to join forces, with families and between HCPs, beyond the boundaries of the original healthcare condition for shared learning. Ultimately, advancing integrated physical, socioemotional and neurocognitive care for these children and their families contributes not only to event-free survival but also, more importantly, to quality of life and true participation in society, the ultimate goal of optimal quality of care.

## Data Availability

The datasets generated during and/or analyzed during the current study are available from the corresponding author on reasonable request.

## References

[1] Long DA, Fink EL. Transitions from short to long-term outcomes in pediatric critical care: considerations for clinical practice. Transl Pediatr. 2021;10:2858–74. 10.21037/tp-21-61. PMC8578758 34765507

[2] Neidenbach R, Achenbach S, Andonian C, Bauer UMM, Ewert P, Freilinger S, et al. Systematic assessment of health care perception in adults with congenital heart disease in Germany. Cardiovasc Diagn Ther. 2021;11:481–91. 10.21037/cdt-20-825. PMC8102265 33968627

[3] de Kloet LC, Bense JE, van der Stoep M, Louwerens M, von Asmuth EGJ, Lankester AC, et al. Late endocrine effects after hematopoietic stem cell transplantation in children with nonmalignant diseases. Bone Marrow Transpl. 2022;57:1564–72. 10.1038/s41409-022-01755-x. 35840745

[4] Patel RM. Short- and long-term outcomes for extremely preterm infants. Am J Perinatol. 2016;33:318–28. 10.1055/s-0035-1571202. PMC4760862 26799967

[5] Raissadati A, Haukka J, Patila T, Nieminen H, Jokinen E. Chronic disease burden after congenital heart surgery: a 47-year population-based study with 99% follow-up. J Am Heart Assoc. 2020;9:e015354. 10.1161/JAHA.119.015354. PMC7428561 32316818

[6] Chong LSH, Fitzgerald DA, Craig JC, Manera KE, Hanson CS, Celermajer D, et al. Children’s experiences of congenital heart disease: a systematic review of qualitative studies. Eur J Pediatr. 2018;177:319–36. 10.1007/s00431-017-3081-y. 29327140

[7] Morgan AS, Mendonca M, Thiele N, David AL. Management and outcomes of extreme preterm birth. BMJ. 2022;376:e055924. 10.1136/bmj-2021-055924. PMC8744861 35012942

[8] Bense JE, Ter Welle L, Mekelenkamp H, Schimmel M, Louwerens M, Lankester AC, et al. Exploring the long-term psychosocial impact of paediatric haematopoietic stem cell transplantation for nonmalignant diseases. Health Expect. 2022;25:2462–70. 10.1111/hex.13565. PMC9615066 35906795

[9] Cheong JLY, Haikerwal A, Anderson PJ, Doyle LW. Outcomes into adulthood of infants born extremely preterm. Semin Perinatol. 2021;45:151483. 10.1016/j.semperi.2021.151483. 34482961

[10] Mekelenkamp H, de Vries M, Saalmink I, Nur E, Kerkhoffs JL, Heijboer H, et al. Hoping for a normal life: decision-making on hematopoietic stem cell transplantation by patients with a hemoglobinopathy and their caregivers. Pediatr Blood Cancer. 2024;71:e30808. 10.1002/pbc.30808. 38082534

[11] Areias ME, Pinto CI, Vieira PF, Teixeira F, Coelho R, Freitas I, et al. Long term psychosocial outcomes of congenital heart disease (CHD) in adolescents and young adults. Transl Pediatr. 2013;2:90–8. 10.3978/j.issn.2224-4336.2013.06.02. PMC4728933 26835299

[12] Barrera M, Atenafu E, Hancock K. Longitudinal health-related quality of life outcomes and related factors after pediatric SCT. Bone Marrow Transpl. 2009;44:249–56. 10.1038/bmt.2009.24. 19234504

[13] Harrison DJ, Kay J, Jacobsen RM, Londono-Obregon C, Yeung E, Kelly SL, et al. The burden of psychological trauma and post-traumatic stress disorder among adults with congenital heart disease: PTSD in ACHD. Am J Cardiol. 2024;219:9–16. 10.1016/j.amjcard.2024.03.007. 38458583

[14] Mamasoula C, Pennington L, Adesanya AM, Rankin J. A systematic review and meta-analysis of school and cognitive function domains of health-related quality of life measures for children and young adults with congenital heart disease. Birth Defects Res. 2024;116:e2275. 10.1002/bdr2.2275. 38014517

[15] van Klink JMM, van Zwet EW, Lopriore E, Roest AAW, Haak MC, Slaghekke F, et al. Does catch-up growth come with a cognitive cost? Cognitive outcome and growth patterns in growth discordant identical twins. J Pediatr. 2024;275:114223. 10.1016/j.jpeds.2024.114223. 39097263

[16] Tollenaar LSA, Lopriore E, Slaghekke F, Oepkes D, Middeldorp JM, Haak MC, et al. High risk of long-term neurodevelopmental impairment in donor twins with spontaneous twin anemia-polycythemia sequence. Ultrasound Obstet Gynecol. 2020;55:39–46. 10.1002/uog.20846. 31432580

[17] Hordijk JA, Verbruggen SC, Buysse CM, Utens EM, Joosten KF, Dulfer K. Neurocognitive functioning and health-related quality of life of children after pediatric intensive care admission: a systematic review. Qual Life Res. 2022;31:2601–14. 10.1007/s11136-022-03124-z. PMC9356943 35357629

[18] Tsao PC, Lee YS, Jeng MJ, Hsu JW, Huang KL, Tsai SJ, et al. Additive effect of congenital heart disease and early developmental disorders on attention-deficit/hyperactivity disorder and autism spectrum disorder: a nationwide population-based longitudinal study. Eur Child Adolesc Psychiatry. 2017;26:1351–9. 10.1007/s00787-017-0989-8. 28417257

[19] Jansen L, Steggerda S, Rijken M, van Steenis A, de Vries L, Peeters-Scholte C, et al. Social-emotional and behavioural issues after very preterm birth: parental and teachers experiences. J Child Health Care. 2024;28:277–90. 10.1177/13674935221121881. 35993442

[20] van Beek PE, Leemhuis AG, Abu-Hanna A, Pajkrt E, Aarnoudse-Moens CSH, van Baar AL, et al. Preterm birth is associated with lower academic attainment at age 12 years: a matched cohort study by linkage of population-based datasets. J Pediatr. 2022;251:60–6.e3. 10.1016/j.jpeds.2022.07.049. 35944725

[21] Oster ME, Watkins S, Hill KD, Knight JH, Meyer RE. Academic outcomes in children with congenital heart defects: a population-based cohort study. Circ Cardiovasc Qual Outcomes. 2017;10:e003074. 10.1611/CIRCOUTCOMES.116.003074. PMC5328588 28228450

[22] Maas A, Maurice-Stam H, Kremer LCM, van der Aa-van Delden A, van Dulmen-den Broeder E, Tissing WJE, et al. Psychosocial outcomes in long-term Dutch adult survivors of childhood cancer: the DCCSS-LATER 2 psycho-oncology study. Cancer. 2023;129:2553–67. 10.1002/cncr.34795. 37057358

[23] de Proost L, de Boer A, Reiss IKM, Steegers EAP, Verhagen AAE, Hogeveen M, et al. Adults born prematurely prefer a periviability guideline that considers multiple prognostic factors beyond gestational age. Acta Paediatr. 2023;112:1926–35. 10.1111/apa.16866. 37272253

[24] Perez A, Thiede L, Ludecke D, Ebenebe CU, von dem Knesebeck O, Singer D. Lost in transition: health care experiences of adults born very preterm-a qualitative approach. Front Public Health. 2020;8:605149. 10.3389/fpubh.2020.605149. PMC7793891 33425841

[25] Wiener L, Sannes TS, Randall J, Lahijani S, Applebaum AJ, Gray TF, et al. Psychosocial assessment practices for hematopoietic stem cell transplantation: a national survey study. Bone Marrow Transpl. 2023;58:1314–21. 10.1038/s41409-023-02087-0. PMC10967240 37634015

[26] Vaitkeviciene GE, Azukaitis K, Jankauskiene A, Petrene J, Puronaite R, Trinkunas J, et al. Development and integration of patient-reported measures into E-health system: pilot feasibility study. Healthcare (Basel). 2023;11:2290. 10.3390/healthcare11162290. PMC10454584 37628488

[27] Kidanemariam M, Pieterse AH, van Staalduinen DJ, Bos WJW, Stiggelbout AM. Does value-based healthcare support patient-centred care? a scoping review of the evidence. BMJ Open. 2023;13:e070193. 10.1136/bmjopen-2022-070193. PMC10335496 37429675

[28] Edgman-Levitan S, Schoenbaum SC. Patient-centered care: achieving higher quality by designing care through the patient’s eyes. Isr J Health Policy Res. 2021;10:21. 10.1186/s13584-021-00459-9. PMC7934513 33673875

[29] Clay AM, Parsh B. Patient- and family-centered care: it’s not just for pediatrics anymore. AMA J Ethics. 2016;18:40–4. 10.1001/journalofethics.2016.18.1.medu3-1601. 26854635

[30] Hsu C, Gray MF, Murray L, Abraham M, Nickel W, Sweeney JM, et al. Actions and processes that patients, family members, and physicians associate with patient- and family-centered care. BMC Fam Pract. 2019;20:35. 10.1186/s12875-019-0918-7. PMC6388493 30803446

[31] Seniwati T, Rustina Y, Nurhaeni N, Wanda D. Patient and family-centered care for children: a concept analysis. Belitung Nurs J. 2023;9:17–24. 10.33546/bnj.2350. PMC10353635 37469640

[32] Teisberg E, Wallace S, O’Hara S. Defining and implementing value-based health care: a strategic framework. Acad Med. 2020;95:682–5. 10.1097/ACM.0000000000003122. PMC7185050 31833857

[33] Nederlandse Federatie van Universitair medische centra (NFU). Position paper waardegedreven zorg. https://nfukwaliteit.nl/pdf/NFU-Position_Paper_Waardegedreven_Zorg.pdf. Accessed 11 Oct 2025.

[34] Bele S, Teela L, Zhang M, Rabi S, Ahmed S, van Oers HA, et al. Use of patient-reported experience measures in pediatric care: a systematic review. Front Pediatr. 2021;9:753536. 10.3389/fped.2021.753536. PMC8721567 34988035

[35] Kingsley C, Patel S. Patient-reported outcome measures and patient-reported experience measures. BJA Educ. 2017;17:137–44. 10.1093/bjaed/mkw060.

[36] Mukherjee S, Richardson N, Beresford B. Hospital healthcare experiences of children and young people with life-threatening or life-shortening conditions, and their parents: scoping reviews and resultant conceptual frameworks. BMC Pediatr. 2023;23:366. 10.1186/s12887-023-04151-6. PMC10351142 37460965

[37] Wray J, Hobden S, Knibbs S, Oldham G. Hearing the voices of children and young people to develop and test a patient-reported experience measure in a specialist paediatric setting. Arch Dis Child. 2018;103:272–9. 10.1136/archdischild-2017-313032. 28903950

[38] Espinel AG, Shah RK, McCormick ME, Krakovitz PR, Boss EF. Patient satisfaction in pediatric surgical care: a systematic review. Otolaryngol Head Neck Surg. 2014;150:739–49. 10.1177/0194599814527232. 24671459

[39] Chatterjee P, Tsai TC, Jha AK. Delivering value by focusing on patient experience. Am J Manag Care. 2015;21:735–7. 26633097

[40] Gooding JS, Cooper LG, Blaine AI, Franck LS, Howse JL, Berns SD. Family support and family-centered care in the neonatal intensive care unit: origins, advances, impact. Semin Perinatol. 2011;35:20–8. 10.1053/j.semperi.2010.10.004. 21255703

[41] Kutahyalioglu NS, Scafide KN. Effects of family-centered care on bonding: a systematic review. J Child Health Care. 2023;27:721–37. 10.1177/13674935221085799. 35430900

[42] Alarilla A, Terrell K, Kelly P, Chesters H, Gibson F, Oldham G, et al. Routine use of patient-reported experience and outcome measures for children and young people: a scoping review. Syst Rev. 2024;13:293. 10.1186/s13643-024-02706-x. PMC11603634 39609878

[43] Gleeson H, Calderon A, Swami V, Deighton J, Wolpert M, Edbrooke-Childs J. Systematic review of approaches to using patient experience data for quality improvement in healthcare settings. BMJ Open. 2016;6:e011907. 10.1136/bmjopen-2016-011907. PMC5013495 27531733

[44] Linnér A, Thernström Blomqvist Y, Jonsson K, Lilliesköld S, Norman M. Parental experiences of neonatal care: a nationwide study on determinants of excellence. Neonatology. 2024;121:46–55. 10.1159/000533900. PMC10836739 37844545

[45] Wray J, Oldham G. Using parent-reported experience measures as quality improvement tools in paediatric cardiothoracic services: making it happen. Int J Qual Health Care. 2020;32:140–8. 10.1093/intqhc/mzaa001. 32159765

[46] Ferreira J, Patel P, Guadagno E, Ow N, Wray J, Emil S, et al. Patient experience or patient satisfaction? a systematic review of child- and family-reported experience measures in pediatric surgery. J Pediatr Surg. 2023;58:862–70. 10.1016/j.jpedsurg.2023.01.015. 36797113

[47] Ang FJL, Finkelstein EA, Gandhi M. Parent-reported experience measures of care for children with serious illnesses: a scoping review. Pediatr Crit Care Med. 2022;23:e416–23. 10.1097/PCC.0000000000002996. 35616499

[48] Heery E, Sheehan AM, While AE, Coyne I. Experiences and outcomes of transition from pediatric to adult health care services for young people with congenital heart disease: a systematic review. Congenit Heart Dis. 2015;10:413–27. 10.1111/chd.12251. 25659600

[49] Sawicki GS, Garvey KC, Toomey SL, Williams KA, Chen Y, Hargraves JL, et al. Development and validation of the adolescent assessment of preparation for transition: a novel patient experience measure. J Adolesc Health. 2015;57:282–7. 10.1016/j.jadohealth.2015.06.004. PMC4548278 26299555

[50] Bull C, Byrnes J, Hettiarachchi R, Downes M. A systematic review of the validity and reliability of patient-reported experience measures. Health Serv Res. 2019;54:1023–35. 10.1111/1475-6773.13187. PMC6736915 31218671

[51] Ebihara A. World medical association declaration of Helsinki. Jpn Pharmacol Ther. 2000;28:983–6. 10.2165/00124363-200010000-00014.

[52] Branchevereniging Integrale KindZorg. PREM kindzorg referentie. https://binkz.nl/over-de-branche/prem-kindzorg/. 11 Oct 2025.

[53] Azukaitis K, Puronaite R, Vaitkeviciene GE, Trinkunas J, Jankauskiene A, Jankauskiene D. Integrated collection of patient-reported outcomes and experiences in children with kidney and hematological diseases: a pilot study. Children (Basel). 2023;10:1245. 10.3390/children10071245. PMC10377830 37508742

[54] Nafees Z, Ferreira J, Guadagno E, Wray J, Anderzén-Carlsson A, Poenaru D. Adaptation, translation, and validation of a patient-reported experience measure for children and young people for the Canadian context. J Pediatr Surg. 2024;59:810–7. 10.1016/j.jpedsurg.2024.01.030. 38369398

[55] Ang FJL, Gandhi M, Ostbye T, Malhotra C, Malhotra R, Chong PH, et al. Development of the parental experience with care for children with serious illnesses (PRECIOUS) quality of care measure. BMC Palliat Care. 2024;23:66. 10.1186/s12904-024-01401-x. PMC10921687 38454420

[56] Bartholdson C, Broström E, Iversen MD, Granhagen JJ. Patient-reported experience measures in pediatric healthcare-a rapid evidence assessment. J Patient Exp. 2024;11:23743735241290480. 10.1177/23743735241290481. PMC11629420 39659439

[57] Sizmur S, Graham C, Bos N. Psychometric evaluation of patient-reported experience measures: is it valid? Int J Qual Health Care. 2020;32:219–20. 10.1093/intqhc/mzaa006. 32103271

[58] McCabe E, Rabi S, Bele S, Zwicker JD, Santana MJ. Factors affecting implementation of patient-reported outcome and experience measures in a pediatric health system. J Patient Rep Outcomes. 2023;7:24. 10.1186/s41687-023-00563-1. PMC9998780 36892738

[59] Braun V, Clarke V. Using thematic analysis in psychology. Qual Res Psychol. 2006;3:77–101. 10.1191/1478088706qp063oa.

[60] Baumgartner H, De Backer J, Babu-Narayan SV, Budts W, Chessa M, Diller GP, et al. 2020 ESC guidelines for the management of adult congenital heart disease. Eur Heart J. 2020;42:563–645. 10.1093/eurheartj/ehaa554. 32860028

[61] Brands MR, Haverman L, Muis JJ, Driessens MHE, van der Meer FJM, Goedhart G, et al. Patients’ and health care providers’ perspectives on quality of hemophilia care in the Netherlands: a questionnaire and interview study. Res Pract Thromb Haemost. 2023;7:100159. 10.1016/j.rpth.2023.100159. PMC10279896 37346462

[62] Hagen IH, Iversen VC, Nesset E, Orner R, Svindseth MF. Parental satisfaction with neonatal intensive care units: a quantitative cross-sectional study. BMC Health Serv Res. 2019;19:37. 10.1186/s12913-018-3854-7. PMC6332571 30646901

[63] Dreyer K, Williamson RAP, Hargreaves DS, Rosen R, Deeny SR. Associations between parental mental health and other family factors and healthcare utilisation among children and young people: a retrospective, cross-sectional study of linked healthcare data. BMJ Paediatr Open. 2018;2:e000266. 10.1136/bmjpo-2018-000266. PMC6069921 30094348

[64] Golfenshtein N, Hanlon AL, Lozano AJ, Srulovici E, Lisanti AJ, Cui N, et al. Parental post-traumatic stress and healthcare use in infants with complex cardiac defects. J Pediatr. 2021;238:241–8. 10.1016/j.jpeds.2021.06.073. PMC8551052 34216630

[65] Thomas S, Ryan NP, Byrne LK, Hendrieckx C, White V. Psychological distress among parents of children with chronic health conditions and its association with unmet supportive care needs and children’s quality of life. J Pediatr Psychol. 2024;49:45–55. 10.1093/jpepsy/jsad074. PMC10799716 37840456

[66] Amin K, Patel K. Role of psychologists in pediatric congenital heart disease. Pediatr Clin North Am. 2022;69:865–78. 10.1016/j.pcl.2022.05.002. 36207098

[67] Kamis C. The long-term impact of parental mental health on children’s distress trajectories in adulthood. Soc Ment Health. 2021;11:54–68. 10.1177/2156869320912520. PMC8172076 34094696

[68] Shaw RJ, Givrad S, Poe C, Loi EC, Hoge MK, Scala M. Neurodevelopmental, mental health, and parenting issues in preterm infants. Children (Basel). 2023;10:1565. 10.3390/children10091565. PMC10528009 37761526

[69] Wade M, Browne DT, Plamondon A, Daniel E, Jenkins JM. Cumulative risk disparities in children’s neurocognitive functioning: a developmental cascade model. Dev Sci. 2016;19:179–94. 10.1111/desc.12302. 25845409

[70] Aleshchenko E, Langer T, Calaminus G, Gebauer J, Swart E, Baust K. Organizing long-term follow-up care for pediatric cancer survivors: a socio-ecological approach. Front Public Health. 2025;13:1524310. 10.3389/fpubh.2025.1524310. PMC11911165 40098799

[71] Ekaterina A, Thorsten L, Gabriele C, Juliane G, Kathrin H, Pietro T, et al. Follow-up care needs and motivational factors for childhood cancer survivors and their parents in Germany. Sci Rep. 2025;15:972. 10.1038/s41598-024-84156-y. PMC11704210 39762346

[72] Taakgroep LATER van de Stichting Kinderoncologie Nederland (SKION). Richtlijn follow-up na kinderkanker: meer dan 5 jaar na diagnose. 2009. https://www.skion.nl/workspace/uploads/richtlijn_follow-up_na_kinderkanker_deel_1_boekje_met_aanbevelingen_1.pdf. Accessed 11 Oct 2025.

[73] van Kalsbeek RJ, van der Pal HJH, Kremer LCM, Bardi E, Brown MC, Effeney R, et al. European PanCareFollowUp recommendations for surveillance of late effects of childhood, adolescent, and young adult cancer. Eur J Cancer. 2021;154:316–28. 10.1016/j.ejca.2021.06.004. 34333209

[74] Marchak JG, Christen S, Mulder RL, Baust K, Blom JMC, Brinkman TM, et al. Recommendations for the surveillance of mental health problems in childhood, adolescent, and young adult cancer survivors: a report from the international late effects of childhood cancer guideline harmonization group. Lancet Oncol. 2022;23:e184–96. 10.1016/S1470-2045(21)00750-6. PMC9639707 35358467

[75] Lawless MT, Tieu M, Golley RK, Kitson AL. Defining life stages and mapping care trajectories: a narrative review of life-course theories, models and frameworks. J Adv Nurs. 2026. 10.1111/jan.70471. PMC13460979 41517947

[76] Halfon N, Forrest CB. The emerging theoretical framework of life course health development. In: Halfon N, Forrest CB, Lerner RM, Faustman EM, editors. Handbook of life course health development. Cham (CH): Springer; 2018. p. 19–43. 31314301

[77] Flierman M, Bossen D, de Boer R, Vriend E, van Nes F, van Kaam A, et al. Parents’ information needs during the first year at home with their very premature born child; a qualitative study. PEC Innov. 2024;4:100270. 10.1016/j.pecinn.2024.100270. PMC10940942 38495319

[78] Phelan R, Rotz S, Dandoy CE, Auletta JJ, Badia P, Bhatt NS, et al. Multicenter study on caregiver experiences in pediatric hematopoietic stem cell transplantation: part II. treatment challenges, communication barriers, and caregiver-driven approaches to mitigation. Transplant Cell Ther. 2025;31:590.e1-16. 10.1016/j.jtct.2025.04.012. 40355027

[79] van Zijl ACM, Obermann-Borst SA, Hogeveen M, Verweij EJTJ, de Vries WB, Geurtzen R, et al. Mind the gap: comparing parents’ information needs about impending preterm birth to current clinical practices using a mixed methods approach. PEC Innov. 2024;4:100297. 10.1016/j.pecinn.2024.100297. PMC11219962 38962499

[80] Witt S, Schuett K, Wiegand-Grefe S, Boettcher J, Quitmann J. Living with a rare disease-experiences and needs in pediatric patients and their parents. Orphanet J Rare Dis. 2023;18:242. 10.1186/s13023-023-02837-9. PMC10422846 37568186

[81] Rijksoverheid. Aanpak basisvaardigheden volwassenen. 2025. https://www.rijksoverheid.nl/onderwerpen/basisvaardigheden/aanpak-basisvaardigheden-volwassenen. Accessed 27 May 2026.

[82] Houtkoop W. Basic skills in the Netherlands: the “literacy” of the Netherlands: economic, social and educational aspects of the language and numeracy skills of the Dutch working population. Amsterdam: Max Goote Knowledge Center for Vocational Education and Adult Education; 1999.

